# A multi-class deep learning model for early lung cancer and chronic kidney disease detection using computed tomography images

**DOI:** 10.3389/fonc.2023.1193746

**Published:** 2023-06-02

**Authors:** Ananya Bhattacharjee, Sameh Rabea, Abhishek Bhattacharjee, Eslam B. Elkaeed, R. Murugan, Heba Mohammed Refat M. Selim, Ram Kumar Sahu, Gamal A. Shazly, Mounir M. Salem Bekhit

**Affiliations:** ^1^ Bio-Medical Imaging Laboratory (BIOMIL), Department of Electronics and Communication Engineering, National Institute of Technology Silchar, Silchar, India; ^2^ Department of Pharmaceutical Sciences, College of Pharmacy, AlMaarefa University, Riyadh, Saudi Arabia; ^3^ Department of Pharmaceutical Sciences, Assam University (A Central University), Silchar, India; ^4^ Microbiology and Immunology Department, Faculty of Pharmacy (Girls); Al-Azhar University, Cairo, Egypt; ^5^ Department of Pharmaceutical Sciences, Hemvati Nandan Bahuguna Garhwal University (A Central University), Tehri Garhwal, India; ^6^ Kayyali Chair for Pharmaceutical Industry, Department of Pharmaceutics, College of Pharmacy, King Saud University, Riyadh, Saudi Arabia

**Keywords:** lung cancer, kidney diseases, computed tomography, modified Xception model, fine-tuning, transfer learning, artificial intelligence

## Abstract

Lung cancer is a fatal disease caused by an abnormal proliferation of cells in the lungs. Similarly, chronic kidney disorders affect people worldwide and can lead to renal failure and impaired kidney function. Cyst development, kidney stones, and tumors are frequent diseases impairing kidney function. Since these conditions are generally asymptomatic, early, and accurate identification of lung cancer and renal conditions is necessary to prevent serious complications. Artificial Intelligence plays a vital role in the early detection of lethal diseases. In this paper, we proposed a modified Xception deep neural network-based computer-aided diagnosis model, consisting of transfer learning based image net weights of Xception model and a fine-tuned network for automatic lung and kidney computed tomography multi-class image classification. The proposed model obtained 99.39% accuracy, 99.33% precision, 98% recall, and 98.67% F1-score for lung cancer multi-class classification. Whereas, it attained 100% accuracy, F1 score, recall and precision for kidney disease multi-class classification. Also, the proposed modified Xception model outperformed the original Xception model and the existing methods. Hence, it can serve as a support tool to the radiologists and nephrologists for early detection of lung cancer and chronic kidney disease, respectively.

## 1 Introduction

Lung cancer is one of the world’s most life-threatening diseases. In 2023, smoking cigarettes will directly cause around 81% lung cancer deaths, with second-hand smoke contributing to an additional 3,560 of the 127,070 fatalities Siegel et al. (1). There are two primary categories of lung nodules: benign and malignant. Lung nodules that are benign remain firmly in their original position and do not spread to other bodily areas Heuvelmans et al. (2). Most benign lung nodules are not malignant. Diet, stress, genetics, local damage, and radiation exposure are among the potential contributory factors to benign tumors Takamori et al. (3). Malignant lung nodules, on the other hand, spread uncontrollably throughout the body through the lymphatic or blood systems Gu et al. (4). A malignant lung tumor needs immediate treatment, and if detected early, the patient may be treated by surgery and chemotherapy. On the other hand, Chronic Kidney Disease (CKD) is a degenerative ailment that affects more than 10% of the world’s population, leading to 800 million individuals (5). Persons with diabetes mellitus and hypertension, older adults and women are more likely to develop CKD. Low- and middle-income nations are particularly burdened by CKD, because they are least prepared to handle its effects Hill et al. (6). It is graded as the 16*^th^* leading cause of death worldwide and is anticipated to move up to 5*^th^* place by 2040 Foreman et al. (7). The most common kidney disorders that impair kidney function are renal cell carcinoma (kidney tumor), cyst development, and nephrolithiasis (kidney stones). A hard object made up of chemicals from the urine constitutes the kidney stone disease Alelign and Petros (8). On the other hand, a kidney cyst consists of fluid within a thin wall that develops on the surface of the kidney Sanna et al. (9). Whereas kidney tumor accounts for the 10 most prevailing cancers worldwide Hsieh et al. (10).

Computed tomography (CT) scan is one of the best methods for examining lung cancer and CKD patients, because it produces images with excellent contrast and provides 3D information Brisbane et al. (11). Due to a global shortage of nephrologists and radiologists, manual inspection of medical images is expensive and time consuming and may result in misdiagnosis. CT images play a crucial part in diagnosing many lung and kidney diseases. Still, the necessity for a second opinion owing to a shortage of healthcare professionals significantly impacts the process. Consequently, early detection of renal problems such as kidney stones, cysts, and tumors and lung diseases appear to be critical in preventing kidney failure Bi et al. (12) and lung cancer Monkam et al. (13).

Traditional healthcare management has its limits, but predictive techniques such as deep learning (DL) algorithms can help to overcome these constraints Chen et al. (14); Singh et al. (15); Krishnamurthy et al. (16); Bhattacharjee et al. (17). The application of DL-based detection may reduce invasive procedures, enhancing the efficacy and sustainability of current healthcare methods Akter et al. (18); Alsuhibany et al. (19); Ardila et al. (20). Nowadays, it is feasible to manage enormous and useful data to enhance lung cancer and CKD diagnosis in decision making by using DL classification algorithms Khan et al. (21); Bhaskar and Manikandan (22); Coudray et al. (23). When healthcare practitioners integrate this information with data from other sources, new solutions using predictive analytics can be developed for early CKD and lung cancer diagnosis, related health concerns, and precision therapy. DL algorithms applied to CT images offer an advantage over conventional techniques in medical image classification scenarios. It eliminates the need for subject expertise by automatically learning high-level features from annotated images. DL techniques contributed to advances in oncology and kidney-related domains by reducing manual interventions. Numerous cancer types, namely, prostate cancer Almeida and Tavares (24), pelvic cancer Kalantar et al. (25) and lung cancer Xie et al. (26) have benefited from DL-based classification algorithms. Therefore, the severe negative effects of CKD and lung cancer on many afflicted people, the global shortage of nephrologists and the onset of AI-based computer-aided diagnosis systems motivated us to propose a DL-based classification model that can assist in early CKD and lung cancer detection.

This paper proposes a DL-based modified Xception model for automatically classifying lung cancer patients and kidney diseases such as cysts, stones, and tumors. The Xception model is modified in such a way that both transfer learning-based pre-trained “imagenet” weights and fine-tuned structure is incorporated into the proposed model and hence, is the novelty of the proposed model. Its main contributions are listed below:

1. An effective multi-class modified Xception model is proposed to ensure the least false positives and negative cases for lung cancer and renal diseases, respectively.
2. The proposed model improved the classification model’s convergence by ensuring no overfitting cases.
3. The proposed model outperformed the existing state-of-the-art techniques.

The remainder of the paper is structured as follows. Section 2 contains the related literature survey. This paper’s materials and methods, including the dataset and the architecture, are described in Section 3. Sections 4 and 5 discuss the obtained results and the inferences drawn from them. Finally, Section 6 presents the Conclusion.

## 2 Related work

This section introduces the various DL architectures used for lung cancer and renal disease classification based on different image modalities. This section is broadly divided into two classes: binary and multi-class classifications of lung cancer and renal diseases, respectively.

A combination of VGG16, AlexNet, and LeNet models was employed for lung cancer diagnosis. The features were best extracted by AlexNet, which was then coupled with the KNN classifier to reach a classification accuracy of 98.74% ToğCheck that all equations and special characters are displayed correctly.açar et al. (27). A cross-residual CNN was employed for binary classification of lung CT images that achieved 92.19% accuracy Lyu et al. (28). A lung tumor identification technique was introduced that employed a deep CNN model for classification and achieved 97.3% accuracy Rani and Jawhar (29). A DL-based binary classification of Squamous Cell Carcinoma (SCC) and Adenocarcinoma (ADC) was performed that achieved AUC of 94.14 and 95.94%, respectively Chen et al. (30). Computer-aided diagnostic approach for determining the possibility of lung nodule malignancy was employed using a SVM classifier and achieved AUC score of 90.05% Gonçalves et al. (31). A novel CNN method was employed for binary classification of lung CT images Asuntha and Srinivasan (32). A maximum intensity projection based CNN model for automatically detecting lung cancer system was introduced that achieved a sensitivity of 92.7% Zheng et al. (33). A radiomics and CNN approach was used for binary classification of SCC and ADC that yielded AUC of 71% Chaunzwa et al. (34). A ResNeXt feature extractor followed by DenseNet classifier was employed for binary lung CT image classification that obtained 93.78% accuracy Zhang et al. (35). A multi-view CNN was introduced for binary classification of lung benign and malignant CT images that attained 90.49% sensitivity Liu and Kang (36).

In lung multiclass classification, Reddy et al. (37) identified Malignant, Normal, and Benign (MNoB) CT images using an advanced CNN model and pre-trained Resnet50 and Xception models. The highest accuracy obtained was 97.40%. Kareem et al. (38) presented a computer vision system for lung cancer identification through five stages, namely, pre-processing, image enhancement, segmentation, feature extraction, and SVM classifier for multi-class classification of MNoB CT images and obtained an accuracy of 89.88%. A. Bhattacharjee et al. (39) compared the performances of DenseNet 121, NASNet Large, and modified EfficientNet networks for MNoB multi-class classification.

In kidney binary classification, an automated DL-based kidney stone detection model was proposed using CT images and obtained an accuracy of 96.82% Yildirim et al. (40). An FCN-based kidney segmentation followed by a fully automatic framework using abdominal CT scans was proposed for kidney cysts detection and achieved a true positive rate of 84.3% Blau et al. (41). A cascaded Convolutional Neural Network (CNN) was proposed for stone detection based on CT images and obtained the highest accuracy of 95% Parakh et al. (42). A morphological cascaded CNN on CT images was proposed for renal lesion detection and obtained an AUC of 87.1% Zhang et al. (43). The presence of kidney tumors in CT images were incorporated using 2D CNN, ResNet 50, and VGG16 of 6, 50, and 16 layers, respectively. The 2D CNN, VGG16, and ResNet 50 achieved accuracy of 97, 60, and 96%, respectively Alzu’bi et al. (44). A residual dual attention-based U-Net model followed by convolution and softmax layer was used for kidney cysts segmentation and classification, respectively. Precision and recall for the model were 96.34 and 96.88%, respectively. Out of a total of 79 CT images, 27 were used as test images Fu et al. (45). An MLP and backpropagation-based ANN was proposed to classify the kidney stones ultrasound images and obtained an accuracy of 98.8% Viswanath and Gunasundari (46). A ResNet-based deep neural network was proposed to distinguish between renal stone and normal CT images and achieved 99.1% Caglayan et al. (47). A 3D U-Net model was used for kidney segmentation followed by a DL-based classification model for kidney stone detection Cui et al. (48). A novel ensembling classifier was proposed for four different types of models such as Bayesian, Decision Tree, ANN, and rule-based classifier. The proposed approach used a genetic algorithm for weight assignment and achieved 97.1% accuracy Kazemi and Mirroshandel (49). Different algorithms such as Random Forest (RF), Decision Tree, Multi-layer perceptron, Naive Bayes, K-Nearest Neighbor, Support Vector Machine (SVM), and CNN were applied to get the best x-ray image classification model for kidney stone and healthy patients. The decision tree model achieved the highest F1 score of 85.3% Aksakalli et al. (50). A conventional and DL transfer learning methods were integrated to feed as input to an SVM classifier, which obtained a maximum of 88% specificity in distinguishing between normal and unhealthy renal ultrasound images Zheng et al. (51).

In kidney multiclass classification, an ensembled deep neural network, consisting of ResNet 101, MobileNet V2, and ShuffleNet networks classified Normal, Cyst, Tumor, and Stone (NCTS) ultrasound images and obtained a maximum multi-class classification accuracy of 96.54% Sudharson and Kokil (52). A VGG19 model was customized by replacing the fully connected layers with a naive inception module and dense layers to classify NCTS CT images Asif et al. (53). This method yielded a classification accuracy of 99.25%. CNN model was employed to classify NCTS CT images and obtained an accuracy of 99.36% Narmada et al. (54). The NCTS CT images classification was performed by first extracting the features through a DenseNet model followed by RF classifier and obtained an accuracy of 99.44% Qadir and Abd (55). Six DL classifiers such as swin transformer, Compact Convolutional Transformer, External Attention Transformer, Inception V3, VGG16, and ResNet were used for NCTS CT images. The maximum accuracy obtained was 99.30% Islam et al. (56).

In summary, various image modalities such as x-ray, ultrasound and CT images were used for lung cancer and renal disease classification. However, the majority of the work is based on binary classification of abnormal and normal images instead of multi-class classification. Considering the gap in the research findings of the above articles and inspired by the work in Bhattacharjee et al. (17), we proposed a fine-tuned and pre-trained transfer learning technique based modified Xception model for automatic multi-class classification of MNoB lung CT images. The present study is also extended for kidney NCTS CT images.

## 3 Materials and methods

The dataset utilized in this study and the proposed improved Xception architecture along with its mathematical equations are covered in detail in this section.

### 3.1 Materials

In this study, two datasets are used, namely, Iraq-Oncology Teaching Hospital/National Center for Cancer Diseases (IQ-OTH/NCCD) Kareem (57) and CT Kidney dataset Islam et al. (56).

**Figure 1:**
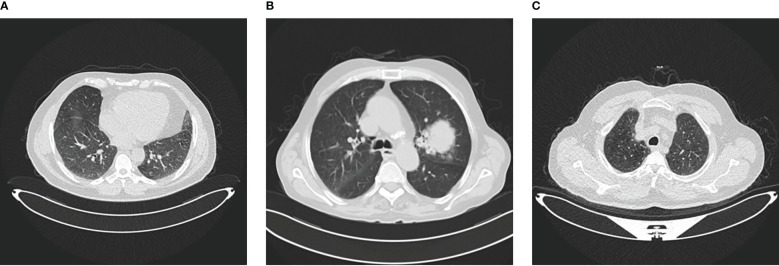
IQ-OTHNCCD dataset **(A)** Benign **(B)** Malignant **(C)** Normal.

The IQ-OTH/NCCD dataset consists of healthy and unhealthy subjects suffering from lung cancer in various stages. This dataset can also be accessed from the Kaggle website Kareem (57). In 2019, the data were gathered for more than 3 months. All of the slides were annotated by the radiologists and oncologists of these two centers. The dataset contains 1,190 images featuring CT scan slices from 110 different instances. The dataset is divided into three categories: MNoB. There are 55 normal cases, 40 malignant cases, and 15 benign cases. Digital Imaging and Communications in Medicine (DICOM) format was used to originally gather the images. However, the JPEG format was subsequently included by the IQ-OTH/NCCD dataset itself. Siemens SOMATOM scanner is employed. One millimeter thick slices are used. The dataset is approved by the institutional review boards of the participating hospitals. Each CT scan is composed of 80 to 200 distinct slices. Every slice is a representation of a distinct angle and side of the human chest. For the 40 malignant instances, there are a total of 561 CT images. The majority of the subjects are from Iraq’s middle area. **Figure 1** shows sample images of each MNoB class.

**Figure 2:**
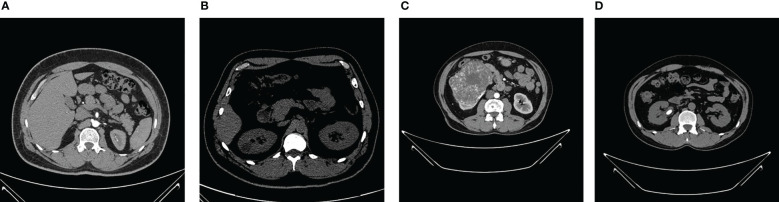
CT Kidney dataset **(A)** Normal **(B)** Cyst **(C)** Tumor **(D)** Stone.

The NCTS dataset was gathered from Dhaka hospital, Bangladesh. Proper consent was taken from all the subjects before collecting the data, which was then approved by Dhaka Central International Medical College and Hospital. The dataset consists of 12,446 total abdomen and urogram CT images, where the axial and coronal cuts were taken. Out of this, the number of NCTS images is 5077, 3709, 2283, and 1377, respectively. The dataset can be accessed from the Kaggle website Islam and Mehedi (58). The dataset was originally in DICOM format, which was later converted to JPEG images through the Sante Dicom editor tool. The CT images were annotated by the Philips IntelliSpace Portal application, which was re-verified by a physician and medical technician to avoid any incorrect annotations. **Figure 2** shows sample images of each NCTS class.

### 3.2 Methodology

**Figure 3:**
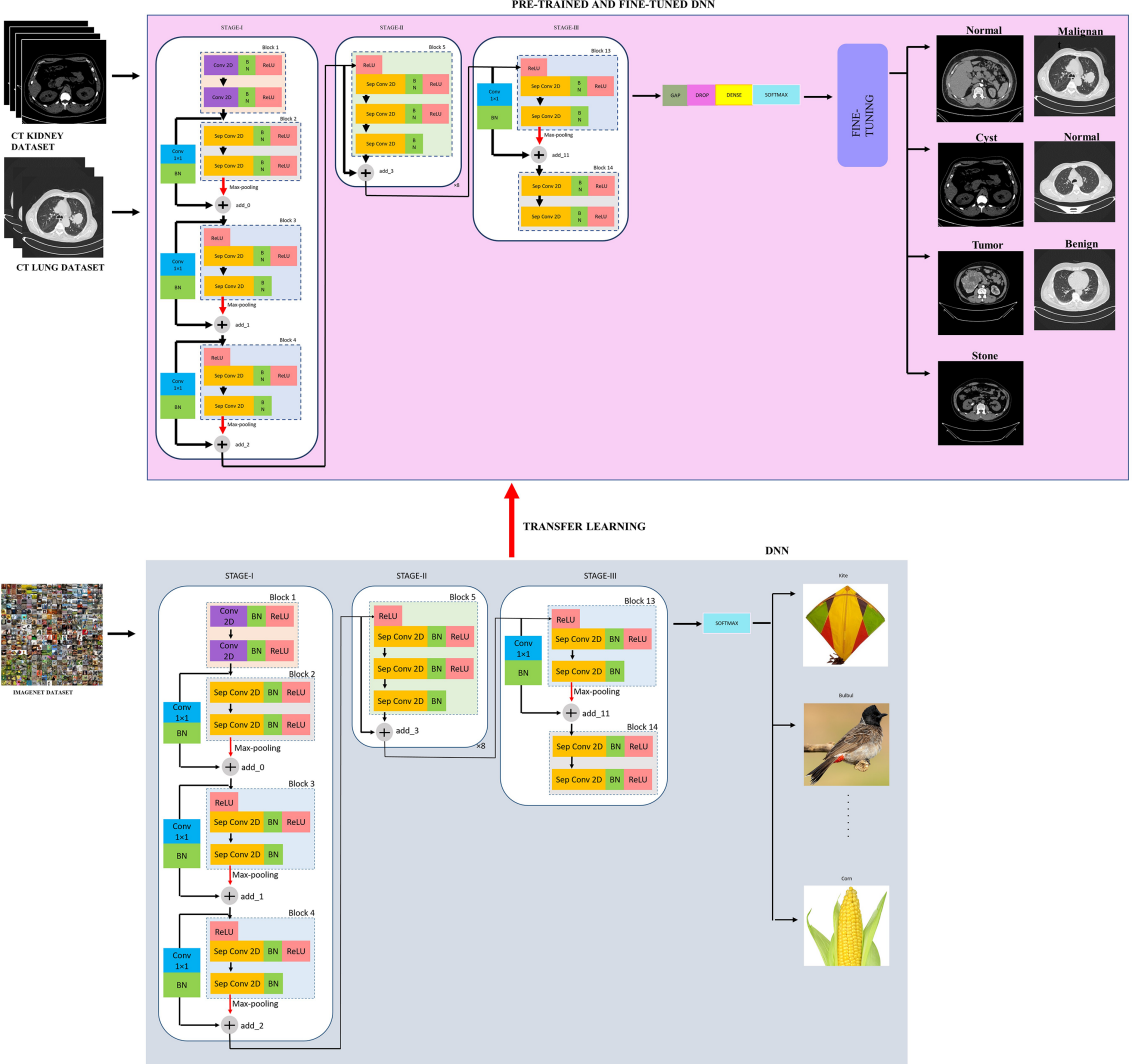
The proposed modified XceptionNet model.

The proposed modified Xception model, short for the “modified Extreme Inception” model, mainly consists of transferring the weights of the “imagenet” to the target network through transfer learning followed by the fine-tuned network, as shown in figure effig:3. The target network is the modified Xception architecture. In the beginning, the input image is first resized from 512×512 to 224×224 during pre-processing for reduced complexity. Then, there are mainly three stages, namely, STAGE-I, STAGE-II, and STAGE-III. First, the source “imagenet” dataset is fed to the original XceptionNet, which is passed over 36 convolutional (conv) layers. Out of these, the number of regular conv and depth-wise Separable Conv 2D layers (Sep Conv 2D) is 2 and 34, respectively. The first block consists of two conv layers of 3×3 kernels, followed by a Batch Normalization (BN) and Rectified Linear Unit (ReLU) activation function. All blocks, except Blocks 1 and 14, contain a linear stack of residually connected Sep Conv 2D layers. The input (I/P) and output (O/P) dimensions determine whether the residual connections are identity or convolution blocks. When the I/P and O/P dimensions are identical, identity mapping is performed, as shown in Block 5 of **Figure 3**. Otherwise, a linear projection is executed using short connections to make the dimensions match each other. Blocks 2, 3, 4, and 13 have a linear projection of conv filter of 1×1 kernel. These blocks also undergo max-pooling operations to extract sharp and smooth features and lower the computing cost by decreasing the amount of parameters that must be learned. BN is included after every conv 2D and Sep Conv 2D layers circumvent the local minima issue by translating the activations to the zero mean and unit variance, hence allowing larger gradient steps for faster convergence Ioffe and Szegedy (59). The use of Sep Conv 2D overcomes the limitation of CNN by segregating the regular conv operations into depth-wise/spatial conv and sequential point-wise conv. This results in fewer parameters compared with regular conv and hence reduces the chances of overfitting Chollet (60). Block 5 is repeated five times having identity mappings. The last block 14 is fed to the softmax layer which finally classifies a thousand classes of imagenet dataset such as kite, bulbul, candle, corn, and so on.

Let the kernel “W” of size 3×3 be convolved with the I/P image “j(*x*, *y*)” and the output “K(*x*, *y*)” is represented by Equation (1), which is then fed to a ReLU activation function “ γ(K) “, given by Equation (2). Similarly, the next Conv 2D outputs “l(*x*, *y*),” given by Equation (3) and then fed to ReLU activation function, given by Equation (4).


(1) k(x,y)=W*j(x,y)

(2) $$\gamma \left(k\right)=\{\begin{array}{l} 0,ifk<0\\ X,ifk\geq 0 \end{array}$$

(3) l(x,y)=W*k(x,y)

(4) $$\gamma \left(l\right)=\{\begin{array}{l} 0,if\;l<0\\ X,if\;l\geq 0 \end{array}$$

Suppose the I/P dimension of the imagenet dataset be of size $D_{p}\times D_{p}\times C$ , where $D_{p}\times D_{p}$ is the I/P image size and C is the number of channels. Let the number of filters/kernels be D of size $D_{q}\times D_{q}\times C$ .

For a regular conv, the output size will be $D_{r}\times D_{r}\times D$ . Let the number of multiplications per conv operation be n. Then the size of the filter will be $D_{q}\times D_{q}\times C$ . As D filters are present and every filter slides horizontally and vertically $D_{r}$ times, the overall number of multiplications is given by Equation (5).


(5) $$\begin{array}{c} Mul_{reg}=D\times D_{r}\times D_{r}\times n\\ =D\times D_{r}^{2}\times D_{r}^{2}\times C \end{array}$$

As Sep Conv 2D is combination of both depth-wise convolutions (d) and point-wise convolutions (p), let the number of “d” multiplications be D and “p” multiplications be P. Thus, the overall multiplications for Sep Conv 2D be given by Equation (6).


(6) $$Mul_{Sep}=D+P$$

The dimension of filters for depth wise operations will be of size $D_{q}\times D_{q}\times 1$ . Considering C channels for the I/P data, the number of such filters needed are “C.” Therefore, the output will be of dimension $D_{r}\times D_{r}\times C$ . Considering all “C” channels, the total multiplications (D) is given by Equation (7).


(7) $$\begin{array}{c} P=\;C\times D_{r}\times D_{r}\times D_{q}\times D_{q}\\ =C\times D_{r}^{2}\times D_{q}^{2} \end{array}$$

In point-wise operation, “C” channels are subjected to 1×1 convolution. Consequently, the filter dimension after this operation will be 1×1×C . For “D” such filters, the output dimension will be $D_{r}\times D_{r}\times D$ . For point-wise convolution, there will be 1×C multiplications. Equation (8) provides the total number of point-wise convolution multiplications.


(8) $$\begin{array}{c} P=C\times D_{r}\times D_{r}\times D\\ C\times D_{r}^{2}\times D \end{array}$$

Thus, the overall multiplications for Sep Conv 2D is given by Equation (9).


(9) $$\begin{array}{c} Mul_{Sep}=C\times D_{r}^{2}\times D_{q}^{2}+C\times D_{r}^{2}\times D\\ =C\times D_{r}^{2}\left(D_{q}^{2}+D\right) \end{array}$$

The relation between the complexity of regular conv and Sep Conv 2D is given by Equation (10).


(10) $$R=\frac{1}{D}+\frac{1}{D_{q}^{2}}$$

Let Q(L) be the desired mapping and the original mapping be indicated by Q(L)+L, executed through shortcut connection followed by addition, as depicted in **Figure 3**.

Since in Block 2, I/P and output dimensions, namely, L and Q, are not equal, their dimensions are matched through a linear projection $S_{A}$ using a shortcut connection. This is shown in Equation (11).


(11) $$y_{Block2}=Q\left(L,\left\{S_{e}\right\}\right)+S_{A}\times L$$

where $Q\left(L,\left\{S_{e}\right\}\right)$ depicts the residual mapping and $S_{e}$ is the weight layer. Ignoring BN for simplicity, $Q=\gamma \left(S_{1}\gamma \left(S_{0}\right)\right)$ where γ indicates ReLU.

Similarly, the outputs of Block 3 and 4 are represented by Equations (12) and (13), respectively.


(12) $$y_{Block3}=Q\left(y_{Block2},\left\{S_{f}\right\}\right)+S_{B}\times y_{Block2}$$

(13) $$y_{Block4}=Q\left(y_{Block3},\left\{S_{g}\right\}\right)+S_{C}\times y_{Block3}$$

Now, the I/P and O/P dimensions from Block 5 to Block 12 are same. Hence, identity mapping is performed by shortcut connections, as shown in Equation (14). In this case, $Q=S_{2}(\gamma \left(S_{1}\gamma \left(S_{0}L\right)\right)$ .


(14) $$\begin{array}{c} y_{Block5}=Q\left(y_{Block4},\left\{S_{r}\right\}\right)+y_{Block4}\\ y_{Block6}=Q\left(y_{Block5},\left\{S_{i}\right\}\right)+y_{Block5}\\ y_{Block12}=Q\left(y_{Block11},\left\{S_{o}\right\}\right)+y_{Block11} \end{array}$$

The output of Block 13 is given by Equation (15).


(15) $$y_{Block13}=Q\left(y_{Block12},\left\{S_{p}\right\}\right)+S_{D}\times y_{Block12}$$

Now, the weights of the imagenet dataset trained on the original XceptionNet is transferred to the target network, which consists of customized top layers and fine-tuned blocks, thus assembling a transfer learning and fine-tuned based modified Xception model. As a result of transfer learning, the input CT kidney dataset and lung IQ-OTH/NCCD dataset fed through XceptionNet acts as feature extractors and the Block 14 is fed to our own customized top layers, which consists of Global Average Pooling (GAP) layers, Dropout (DROP) layer having dropout ratio of 0.05, 4, and 3 dense layers for NCTS and MNoB, respectively. Last, a softmax activation function is applied. Then, fine-tuning is performed by unfreezing the top 20 layers leaving BN layers frozen. If BN layers are set as trainable, the first epoch following unfreezing will result in a considerable reduction in accuracy. Thus, an efficient modified Xception approach is proposed that ensures higher performance in effective multi-class classification of NCTS and MNoB CT images. **Table 1** explains the proposed architecture in detail.

**Table 1:** Architecture detail of the proposed model.

Stage	Block/Op	Layer Type	F/P size	S/D	O/P shape
I/P	–	–	–	–	(224 × 224 × 3)
Stage I	Block 1	Conv 1	3×3, 32	2	(112 × 112 × 32)
		Conv 2	3×3, 64	1	(109 × 109 × 64)
	Block 2	Sep Conv 1	3×3, 128		(109 × 109 × 128)
		Sep Conv 2	3×3, 128	1	(109 × 109 × 128)
	Maxpooling	–	2×2	–	(55 × 55 × 128)
		Conv	1×1, 128	2	(55 × 55 × 128)
	add_0	–	–	–	(55 × 55 × 128)
	Block 3	Sep Conv 1	3×3, 256	1	(55 × 55 × 256)
		Sep Conv 2	3×3, 256	1	(55 × 55 × 256)
	Maxpooling	–	2×2	–	(28 × 28 × 256)
		Conv	1×1, 256	2	(28 × 28 × 256)
	add_1	–	–	–	(28 × 28 × 256)
	Block 4	Sep Conv 1	3×3, 728	1	(28 × 28 × 728
		Sep Conv 2	33, 728	1	(28 × 28 × 728
	Block 4	–	2×2	–	(14 × 14 × 728)
		Conv	1×1, 728	2	(14 × 14 × 728)
	add_2	–	–	–	(14 × 14 × 728)
Stage II (repeated 8 times)	Block 5	Sep Conv 1	3 × 3, 728	1	(14 × 14 × 728)
		Sep Conv 2	3 × 3, 728	1	(14 × 14 × 728)
		Sep Conv 3	3 × 3, 728	1	(14 × 14 × 728)
	add_3	–	–	–	(14 × 14 × 728)
Stage III	Block 13	Sep Conv 1	3 × 3, 728	1	(14 × 14 × 728)
		Sep Conv 2	3 ×3, 1024	1	(14 × 14 × 1024)
	Maxpooling	–	2×2	–	(7 × 7 × 1024)
		Conv	1 × 1, 1024	2	(7 × 7 × 1024)
	add_11	–	–	–	(7 × 7 × 1024)
	Block 14	Sep Conv 1	3 × 3, 1536	1	(7 × 7 × 1536)
		Sep Conv 2	3 × 3, 1536	1	(7 × 7 × 2048)
GAP	–	–	–	–	2048
Dropout	–	–	–	0.5	2048
Dense	–	–	–	–	4

### 3.3 Pseudocode

The NCTS and MNoB CT images are fed as input to the proposed model, which yields multi-class classified output. The input image shape is set as 224×224 for both the cases. The batch size, learning rate (lr) and dense layers are set as 12, 0.00001 and 4, respectively. First, all the input image is appended from the NCTS and MNoB image directory. Subsequently, the labels for each of the case are also appended. Since there are four classes in NCTS and three in MNoB, the labels for NCTS ( $l_{NCTS}$ ) range from 0 to 3 and $l_{MNoB}$ range from 0 to 2, respectively. Second, the train test split is maintained in the ratio of 80 to 20%. Third, the Xception model is loaded with “imagenet” weights, and the topmost layers are replaced with GAP, DROP, and four dense layers. Then, fine-tuning is accomplished by defrosting the upper 20 layers while freezing the BN layers. If BN layers are set to trainable, the first epoch after unfreezing will result in a significant accuracy decrease. Later, the modified Xception model is compiled using Adam optimizer, sparse categorical cross-entropy loss function and accuracy metric. This process leads to a multiclassified NCTS and MNoB output. The **Algorithm 1** describes the pseudocode of the proposed model.

Algorithm 1 Pseudocode of the proposed model.
**Input:** NCTS and MNoB CT images
**Output:** Multi-class classified output
**Initialize:** input_shape = (224, 224, 3), batch_size = 12, lr = 0.00001, Dense = 4, GAP: Global Average Pooling, DROP: Dropout, BN: Batch Normalization
**Procedure:** l_NCTS_ = {0, 1, 2, 3}
l_MNoB_ = {0, 1, 2}
for image in image_directory:
data.append(image)
labels.append(l_MNoB_ or l_NCTS_)
X_train, X_test, y_train, y_test = train_test_split (data, labels, test_size = 0.20)X = Xception (weights = “imagenet,” include_top=False, `input_shape=input_shape)
model.fc ← GAP, DROP, Dense
model ← Model(inputs = X.inputs, outputs = model.fc)
opt = Adam(lr = 0.00001)
for layer in model.layers[-20:]:
if not instance(layer, layers.BN):
layer.trainable = True
model.compile(optimizer=opt, loss = “sparse_categorical_crossentropy”, metrics=[“accuracy”])
end

## 4 Results

This section comprises three subsections, namely, implementation detail, experimental setup, and experimental results.

### 4.1 Implementation detail

Google colab pro plus was used for executing the proposed model, whose specifications are listed below. The python, keras and tensorflow version used are 3.8.10, 2.9.0 and 2.9.2, respectively. The GPU “NVIDIA A100-SXM” of 11.6 CUDA version and 83.48 GB RAM are used.

### 4.2 Experimental setup

The I/P RGB images of shape 224×224×3 were divided into 80% training and 20% testing data. An experiment consisting of four networks, namely, Inception ResNet V2, Inception V3, NASNet and the proposed network, was conducted to get the bestNCTS model. The proposed model was trained with a batch size of 12 and an “Adam” optimizer with a learning rate equal to 0.00001 was used. The training was terminated *via* an early ending callback if the validation loss does not improve after nine epochs. The topmost layers of these networks were replaced with customized layers. Except the proposed model, all other networks were replaced with top layers having 2,048, 1024, and 512 dense layers followed by the ReLU activation function and four dense layers followed by the softmax activation function. Whereas, the proposed modified Xception model was replaced with GAP, DROP of 50% and four dense layers followed by a softmax activation function.

Similarly, an experiment was conducted for MNoB multi-class classification among the proposed model, Inception ResNet V2, Inception V3 and MobileNet V3 Small. The batch size, optimizer, learning rate, early stopping callback criteria and top layers all are kept the same as the NCTS case except three dense layers are used for MNoB classification instead of four.

The performance metrics such as Accuracy (Train, Test, and Validation), Precision, Recall and F1 score were used for evaluating the proposed modified Xception model and other pre-trained networks. Accuracy (Acc) describes closeness between the positively anticipated value and the actual samples, represented by Equation (16). Precision (Pr) is the percentage of correctly anticipated positives. It is given by Equation (17). Recall (Re) determines the fraction of anticipated positives that are accurate, givenby Equation (18). F1-score is the weighted harmonic mean of Re and Pr, represented by Equation (19).


(16) $$Acc=\frac{TruPos+TruNeg}{TruPos+TruNeg+FalPos+FalNeg}$$

where TruPos, TruNeg, FalPos, and FalNeg represent True Positives, True Negatives, False Positives, and False Negatives, respectively, and Acc represents Accuracy.


(17) $$Prec=\frac{TruPos}{TruPos+FalPos}$$

(18) $$Rec=\frac{TruPos}{TruPos+FalNeg}$$

(19) $$F1-score=\frac{2\times Pr\times Re}{Pr+Re}$$

### 4.3 Experimental result

**Table 2** shows the Train Acc, Validation Acc, and Test Acc for different models, such as Inception ResNet V2, Inception V3, NASNet Large, and the proposed modified Xception model. Inception ResNet V2 achieved least training, validation and testing accuracy of 40.79, 40.81, and 40.81%, respectively. Whereas, the proposed Xception model attained the maximum Train, Validation and Test Acc of 99.79, 99.92, and 100%, respectively.

**Table 2:** Train, Validation, and Test Accuracy for different models of kidney NCTS dataset.

Model	Train Acc (%)	Validation Acc (%)	Test Acc (%)
Inception ResNet V2	40.79	40.81	40.81
Inception V3	97.24	97.91	98.82
NASNet Large	98.04	98.77	99.30
**The proposed Xception model**	**99.79**	**99.92**	**100**

Bold values represent best values obtained from the comparison by the proposed model.

**Table 3** shows the performance metrics of different models for NCTS classification. Inception ResNet V2 obtained 0% Pr, Re, and F1-score for normal, tumor, and stone classes whereas, it obtained 41% precision, 100% recall and 58% F1-score for kidney cyst class. Thus, it means that it is inefficient in distinguishing among normal, tumor, and stone classes. On the otherhand, NASNet Large and Inception V3 outperformed the Inception ResNet V2 model. However, the proposed modified Xception model obtained the best results by achieving 100% Pr, Re, and F1-score for each of the NCTS classes.

**Table 3:** Performance metrics of different models for NCTS classification.

Metrics	Precision (%)				Recall (%)				F1-score (%)			
Models	N	C	T	S	N	C	T	S	N	C	T	S
Inception ResNet V2	0	41	0	0	0	100	0	0	0	58	0	0
Inception V3	98	100	96	99	99	99	98	99	99	99	97	99
NASNet Large	99	99	100	100	99	100	99	98	99	100	99	99
**Proposed model**	**100**	**100**	**100**	**100**	**100**	**100**	**100**	**100**	**100**	**100**	**100**	**100**

Bold values represent best values obtained from the comparison by the proposed model.

**Figure 4:**
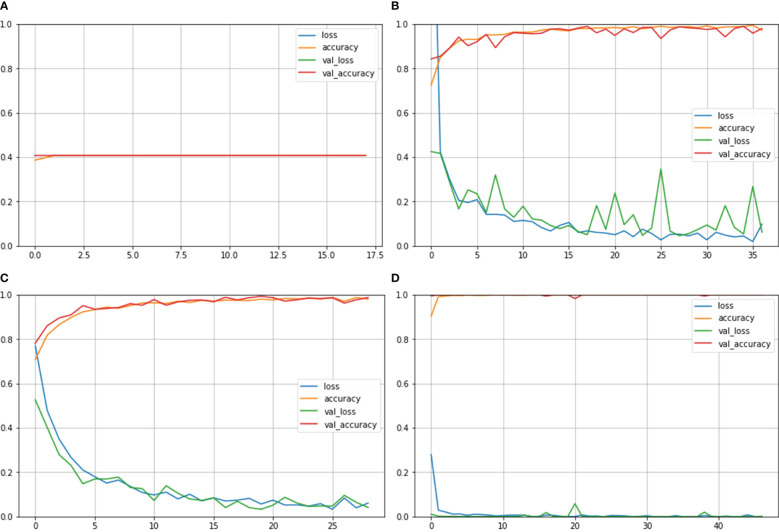
Train Acc, Validation Acc, Training loss, Validation loss curves of NCTS dataset **(A)** Inception ResNet V2 **(B)** Inception V3 **(C)** NASNet Large **(D)** Proposed model.

**Figure 4** depicts the accuracy and loss curves for all the four different models mentioned above. Inception ResNet V2 showed the poorest accuracy and loss curves, showing accuracy of approximately 40%. The Inception V3 and NASNet Large showed training and validation accuracy of less than 100% and training and validation loss above 0%. However, the proposed model achieved the Train and Validation Acc of 100% and loss of 0%. Also, the epochs for the various NCTS models are not constant because an early termination callback was triggered if the validation loss did not decrease after 9 iterations of training.

**Figure 5:**
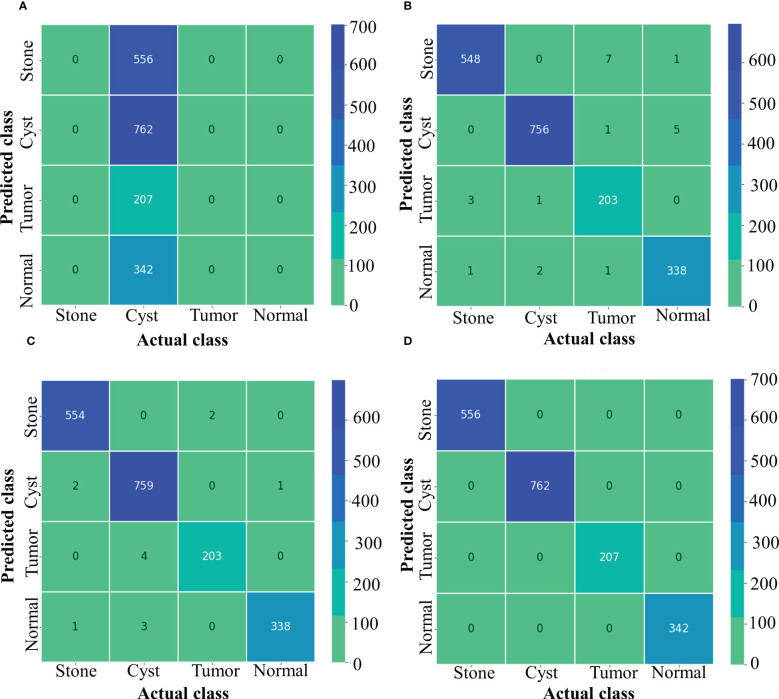
Confusion matrix of NCTS **(A)** Inception resnetv2 **(B)** Inception v3 **(C)** NASNet Large **(D)** Proposed model.

**Figure 5** depicts confusion matrix for each model. Since the Inception ResNet V2 achieved the poorest results, thus, it is unable to detect any other class except kidney cyst. Moreover, it misclassified other classes and considered them to be part of cyst class, whereas NASNet Large showed less misclassification classes than Inception V3. However, the proposed model showed no misclassification results and perfectly classified NCTS classes. Therefore, the proposed model is superior to the existing models for multi-class classification of NCTS classes.

**Table 4** depicts the Train, Validation, and Test Acc of different models such as Inception ResNet V2, Inception V3, MobileNet V3 Small, and the proposed model for MNoB classes. Inception ResNet V2 obtained the least Train, Validation and Test Acc of 51.18, 50.91, and 50.91%, respectively. Although Inception V3 and MobileNet V3 Small achieved almost same training and validation accuracy, their testing accuracies are different. MobileNet achieved approximately 2% lower test accuracy than Inception V3, whereas the proposed modified Xception model obtained the highest Train, Validation, and Test Acc of 100, 99.39, and 99.39%, respectively. **Table 5** shows the evaluation metrics such as Pr, Re and F1 score of the above mentioned models for each MNoB class. Inception ResNet V2 obtained 51% Pr, 100% Re and 67% F1-score for normal class. It is unable to detect malignant and benign class, thus, proving it to be inefficient. Inception V3 and MobileNet V3 Small performed better than Inception ResNet V2, whereas the proposed model obtained the highest Pr, Re, and F1-score of 100, 100, 98, 94, 100, 100, 97, 100, and 99%, respectively for each MNoB class.

**Table 4:** Train, Validation and Test Accuracy for different models of lung cancer dataset.

Models	Training Acc (%)	Validation Acc (%)	Testing Acc (%)
Inception ResNet V2	51.18	50.91	50.91
Inception V3	98.18	93.33	96.36
MobileNet V3 Small	98.39	93.33	94.54
**Proposed model**	**100**	**99.39**	**99.39**

Bold values represent best values obtained from the comparison by the proposed model.

**Table 5:** Performance metrics of different models for MNoB classification.

Metrics	Precision (%)			Recall (%)			F1-score (%)		
Models	M	No	B	M	No	B	M	No	B
Inception ResNet V2	0	51	0	0	100	0	0	67	0
Inception V3	100	95	97	78	99	98	88	97	98
MobileNet V3 Small	92	100	78	94	99	78	93	99	78
**Proposed model**	**100**	**100**	98	**94**	**100**	100	**97**	**100**	99

Bold values represent best values obtained from the comparison by the proposed model.

**Figure 6:**
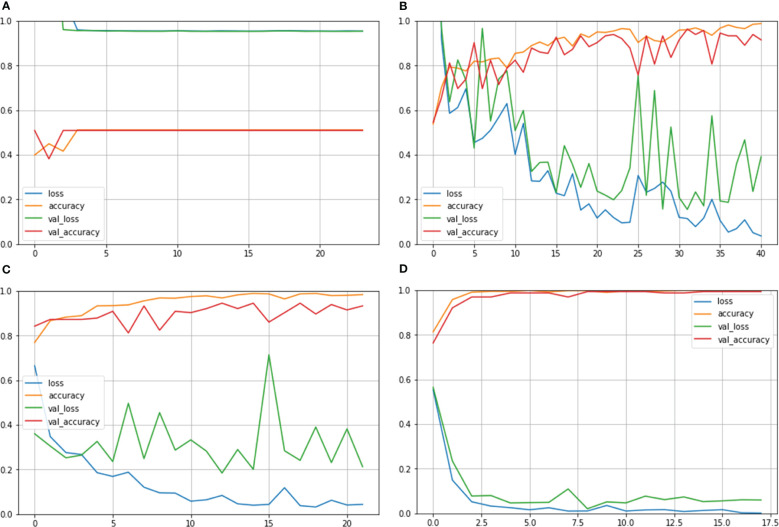
Train Acc, Validation Acc, Train loss, Validation loss curves of lung cancer MNoB dataset **(A)** Inception ResNet V2 **(B)** Inception V3 **(C)** MobileNet V3 Small **(D)** Proposed model.

**Figure 6** shows the Train Acc, Validation Acc, Train loss and Validation loss curves of the different models and the proposed model for MNoB classes. Inception ResNet V2 showed the poorest accuracy and loss curves, showing accuracy of approximately 50% and sparse categorical cross entropy loss of 0.9543. Inception V3 and MobileNet V3 fits the training data too closely but unable to generalize well on testing data, thus showing the sign of overfitting. However, the proposed Xception model obtained the highest Train Acc of 100% and sparse categorical cross entropy loss of 0.0015. Thus, the training loss curves obtained by NCTS model is better than the MNoB because of the reduced gap between the training and validation curves and, hence, it validates that there are no overfitting cases.

**Figure 7:**
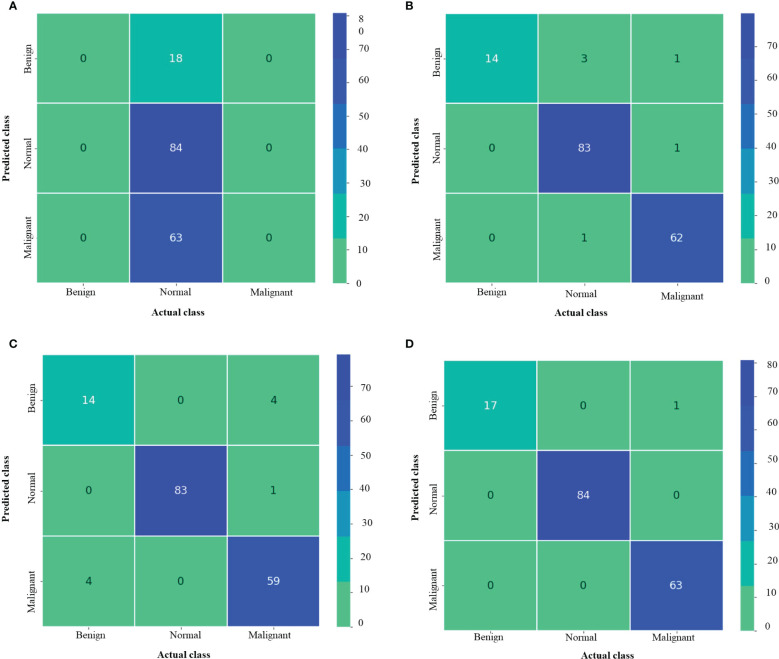
Confusion matrix of MNoB **(A)** Inception resnetv2 **(B)** Inception v3 **(C)** MobileNet V3 Small **(D)** Proposed model.

**Figure 7** depicts the confusion matrix for each of the MNoB models. Since the Inception ResNet V2 achieved the poorest results, thus, it is unable to detect any other class except normal. Moreover, it misclassified other classes and considered them to be part of normal class. Whereas Inception V3 showed less misclassification classes than MobileNet V3 small. However, the proposed model showed only one misclassification result by considering malignant class to be benign. Otherwise, it perfectly classified MNoB classes. Therefore, the proposed model is superior to existing models for multi-class classification of MNoB classes.

## 5 Discussion

A highly effectual renal and lung cancer disease multi-class classifier aid in its early detection and reduces the chances of kidney failure and mortality rate of the affected patients, respectively. However, detecting renal ailments at the precise location in the CT images is challenging due to scarcity of nephrologists availability worldwide, especially in low-income countries Osman et al. (61). Also, manually detecting lung cancer from the CT images is laborious and error prone. Over the years, researchers have conducted substantial study on automatic renal and lung cancer disease classification, but mostly on binary classes. The present study overcomes this research gap by focusing on multi-class classification of several kidney ailments and lung cancer, respectively. The robustness test of the proposed model is conducted by comparing the proposed model with the original Xception model in terms of evaluation metrics and the state-of-the-art techniques.

The following are the key findings of the proposed model:

1. The modified Xception model outperforms the original Xception model in terms of computational time and evaluation metrics such as Average (Avg) Pr, Avg Re, Avg F1 score and test accuracy. The average is found out by taking the mean of all the four NCTS classes.

2. The proposed architecture surpasses the other pre-trained networks, namely, Inception ResNet V2, NASNet Large and Inception V3 in case of NCTS classification. Similarly, it outperforms the Inception ResNet V2, Inception V3 and MobileNet V3 Small in case of MNoB classification.

3. Invoking fine-tuning into the transfer learning based Xception architecture promotes faster training and reduced computational complexity.

4. The proposed model not only performed well on larger NCTS dataset containing approx. 12,000 CT images, but it performed good even in the case of smaller MNoB dataset containing approximately 1,200 images. Thus, the proposed model is a robust model.

5. The proposed model provides edge over performance to the state-of-the-art techniques.

### 5.1 Comparison of computational time between the proposed model and the original Xception model

**Figure 8:**
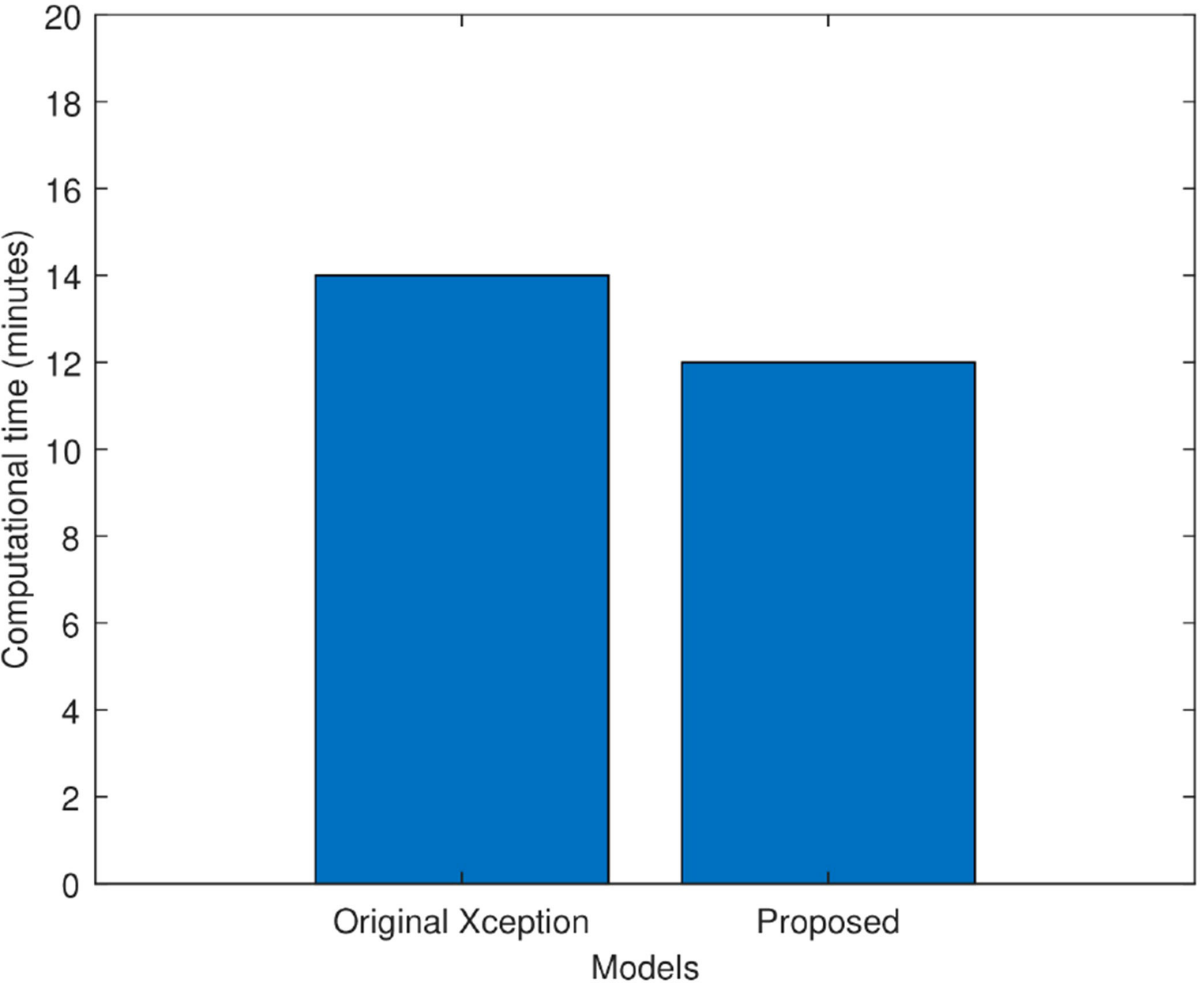
Computational time comparison of the original Xception model and the proposed Xception model in minutes.

**Figure 8** compares the proposed improved Xception model’s computing time to that of the original Xception model in minutes. It is observed that the original Xception network took 14 min to train itself. Whereas, the proposed model took 12 min to train itself. Hence, fine tuning helped the proposed model in faster training and hence, reduced its computational complexity.

### 5.2 Comparison of the proposed model with the original Xception model

**Table 6** compares the proposed modified Xception model with the original Xception model in terms of performance metrics for both NCTS and MNoB cases. In NCTS case, the original model obtained 94.75% Average (Avg) precision, 97.5% Avg recall, 95.75% Avg F1 score and 97.07% test accuracy. On the other hand, the proposed model attained 100% of each of the performance metrics value. In MNoB case, the original Xception model obtained 58.33% Avg Pr, 65.33% Avg Re, 61.33% Avg F1 score and 87.27% Test Acc, whereas the proposed model obtained 99.33% Avg Pr, 98% Avg Re, 98.67% Avg F1 score and 99.39% Test Acc. Thus, it can be inferred that the original Xception model performed poorer for MNoB case compared with NCTS case, whereas, the proposed model performed equally good on both NCTS and MNoB cases. Hence, the proposed Xception model is appropriate for renal disease classification.

**Table 6:** Comparison of the proposed modified Xception model with the original Xception model.

Type	Models	Avg Pr (%)	Avg Re (%)	Avg F1 score (%)	Test Acc (%)
NCTS	Original Xception	94.75	97.5	95.75	97.07
	**Proposed modified Xception**	**100**	**100**	**100**	**100**
MNoB	Original Xception	58.33	65.33	61.33	87.27
	**Proposed modified Xception**	**99.33**	**98**	**98.67**	**99.39**

Bold values represent best values obtained from the comparison by the proposed model.

### 5.3 Comparison of the proposed NCTS model with MNoB in terms of ROC curve

**Figure 9:**
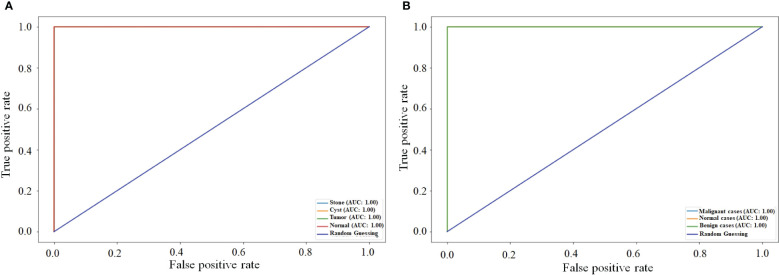
ROC curves **(A)** NCTS **(B)** MNoB.

**Figure 9** compares the ROC curve of both NCTS and MNoB models. Each class of NCTS and MNoB models obtained 100% AUC values. Thus, it is evident that both NCTS and MNoB models performed equally good in terms of ROC curve.

### 5.4 Comparison of the proposed model with the state-of-the-art techniques

The proposed NCTS model is compared with External Attention Transformer (EANET) Islam et al. (56), Compact Convolutional Transformer (CCT) Islam et al. (56), Swin Transformer Islam et al. (56), VGG 16 Islam et al. (56), Inception V3 Islam et al. (56), ResNet 50 Islam et al. (56), DenseNet 201 Qadir and Abd (55), Ensemble DNN Sudharson and Kokil (52), VGG 19 Asif et al. (53) and CNN Narmada et al. (54), as shown in **Table 7**. The proposed model surpassed all the state-of-the-art techniques by achieving the highest Acc, Avg Pr, Avg Re and Avg F1-score of 100% each. Hence, the proposed model is felicitous for automatic CKD diagnosis.

**Table 7:** The proposed NCTS model comparison with the state-of-the-art techniques.

Ref	Models	Acc	Avg Pr	Avg Re	Avg F1-score
Islam et al. (56)	EANET	77.02	81.6	78	77.18
	CCT	96.54	96.52	96.55	96.5
	Swin Transformer	99.30	99.15	99.15	99.12
	VGG16	98.20	98.22	98.12	98.17
	Inception V3	61.60	63.92	62	59.15
	ResNet 50	73.80	73.9	73.75	73.6
Qadir and Abd (55)	DenseNet 201	99.44	99.45	99.47	99.42
Sudharson and Kokil (52)	Ensemble DNN	96.54	96.25	96.5	96.5
Asif et al. (53)	VGG 19	96.37	96.25	96.5	96.5
Narmada et al. (54)	CNN	99.36	99.36	99.38	99.36
**-**	Proposed	**100**	**100**	**100**	**100**

Bold values represent best values obtained from the comparison by the proposed model.

**Table 8** shows the comparison among the proposed MNoB model and the state-of-the-art models such as Advanced CNN Reddy et al. (37), Advanced CNN + Synthetic Minority Oversampling Technique SMOTE) Reddy et al. (37), Xception Reddy et al. (37) and ResNet 50 Reddy et al. (37), SVM Kareem et al. (38), DenseNet 121 Bhattacharjee et al. (39) and NASNet Large Bhattacharjee et al. (39). ResNet 50 Reddy et al. (37) performed the poorest followed by Xception Reddy et al. (37). However, the proposed modified MNoB Xception model outperformed all the state-of-the-art techniques by achieving the highest Acc, Avg Pr, Avg Re, and Avg F1-score of 99.39, 99.33, 98, and 98.67%, respectively.

**Table 8:** The proposed MNoB model comparison with the state-of-the-art techniques.

Ref	Models	Acc	Avg Pr	Avg Re	Avg F1-score
Reddy et al. (37)	Advanced CNN	77.77	72	69	69
	Advanced CNN + SMOTE	97.40	96	97	97
	Xception	81.48	55	63	58
	ResNet 50	76.85	53	59	54
Bhattacharjee et al. (39)	DenseNet 121	95.76	98	92.67	93.67
	NASNet Large	86.67	77.67	79.67	78.33
Kareem et al. (38)	SVM	89.88	97.14	98	97.84
–	**Proposed MNoB**	**99.39**	**99.33**	**98**	**98.67**

Bold values represent best values obtained from the comparison by the proposed model.

### 5.5 Limitation of the proposed model

Despite encouraging results, the proposed MNoB model could not outperform the NCTS model due to the lack of training data. This issue can be solved by implementing data augmentation techniques to expand the training data. Moreover, since the proposed MNoB and NCTS models are two-dimensional, it cannot extract context from adjacent slices. This problem can be solved by introducing a 3D classification model that can take advantage of inter-slice context and, hence, enhance model’s performance.

## 6 Conclusions

In this article, we have proposed a fine tuned and pre-trained transfer learning based modified Xception model for automatic lung cancer and CKD diagnosis. The proposed model can distinguish among benign, normal and malignant lung CT images. Also, it can classify normal, tumor, cyst, and stone multi-classes renal CT images effectively. The proposed methodology composes of mainly four parts, namely, pre-processing, feature extraction, fine tuning, and customized top layers. Pre-processing is performed by resizing the input image from 512×512 to 224×224 for reduced complexity. Feature is extracted using pre-trained “imagenet” weights through Xception based transfer learning technique. The top 20 layers are unfreezed, keeping BN layers frozen to fine-tune the transfer learning based network so that an improved model is obtained. Last, the top most layers are customized using GAP, DROP, DENSE, and softmax layers for improving the potential and generalizibility of the model. The proposed modified Xception MNoB and NCTS model outperformed the existing Xception model and the state-of-the-art techniques. The NCTS model achieved 100% Acc, Pr, Re and F1-score. Whereas, the MNoB model obtained 99.39% Acc, 99.33% Avg Pr, 98% Avg Re, and 98.67% average F1 score. Hence, the proposed model is felicitous for early lung cancer and CKD prediction. It can ensure efficient management of lung cancer and CKD patients by aiding radiologists and nephrologists in diagnosing lung cancer and kidney abnormalities from CT images, respectively. The future scope of this study is that more advanced algorithms such as Squeeze and Excitation, Transformer Block and Dense Block can be incorporated for improved performance. An ensemble of squeeze and excitation, dense block and vision transformer can be implemented for enhanced performance. Also, the training data scarcity in case of MNoB can be addressed through data augmentation techniques or by using generative adversarial network. Moreover, 3D model can be leveraged in future to take into account the inter-slice context.

## Data availability statement

The original contributions presented in the study are included in the article/supplementary material. Further inquiries can be directed to the corresponding authors.

## Author contributions

Conceptualization, AnB, AbB. Methodology, AnB, RM, AbB. Validation, AnB, SR, AbB. Formal analysis, RM, EE, HS. Writing—original draft preparation, AnB, RM, AbB. Writing—review and editing, AnB, AbB, RS, MSB, GS. Supervision, RM. All authors contributed to the article and approved the submitted version.
